# 747 Important Considerations for Healthcare Outreach Missions

**DOI:** 10.1093/jbcr/irae036.290

**Published:** 2024-04-17

**Authors:** Susan L Smith

**Affiliations:** Orlando Health, Orlando, FL

## Abstract

**Introduction:**

Up to 2 million people from the United States participate in short term medical mission trips to over 1000 missions annually with approximately 500 organizations spending $250 million annually in support of these trips. Depending on the mission, a wide variety of volunteers may be needed to support the effort, from multidisciplinary healthcare teams to translators and city planners. Moving past the romantic notion of “doing good”, it is imperative that participants follow a well-organized approach to trip preparation, delivery of care, and establishment of a sustainable plan when departing.

**Methods:**

A review of existing literature was conducting to delineate the process to prepare outreach volunteers and the host country to maximize time and efforts. Key search terms included: medical and international mission, low-middle income country, medical and international outreach, medical volunteer, volunteer abroad. PubMed®, CINAHL®, MedlinePlus®, OVID®, and Google Scholar® were searched.

**Results:**

Pre-trip information for participants should exceed the necessary logistics of finances, telecommunication, vaccinations, and travel arrangements. Preparation should address vital aspects of the host country, such as government structure, income, available medication and healthcare facilities, religion, weather, and available food sources. Sustainability mandates knowledge of and respect for skill set and host resources. Post trip evaluation is essential for participants to debrief about the physical, emotional and educational aspects of the mission. Evaluation by the host country provides critical information for ongoing support and future outreach. Missions should not disrupt local health and social services, but work to achieve productive and foundational outcomes.

**Conclusions:**

Whether public good, private consumption or investment exchange, serious adverse consequences can result when there is poor alignment with host country needs. Research supported that isolated brief missions often do not have any impact on local poverty and struggling healthcare systems. Prioritizing local capacity building through sustainable educational, professional and appropriate financial resources, with recurring support is essential to achieving a long term positive impact. Efforts must avoid creating a culture of dependence, thereby perpetuating a power differential.

**Applicability of Research to Practice:**

With the volume of outreach missions conducted and the large number of organizations and individuals involved in this effort annually, there is an enormous opportunity for research analyzing outcomes at a variety of intervals form the perspective of the volunteers, supporting organizations and the host countries. This data is crucial to refining efforts to truly achieve the greatest good.

